# A Probabilistic Approach to Estimate the Temporal Order of Pathway Mutations Accounting for Intra-Tumor Heterogeneity

**DOI:** 10.3390/cancers16132488

**Published:** 2024-07-08

**Authors:** Menghan Wang, Yanqi Xie, Jinpeng Liu, Austin Li, Li Chen, Arnold Stromberg, Susanne M. Arnold, Chunming Liu, Chi Wang

**Affiliations:** 1 Department of Statistics, University of Kentucky, Lexington, KY 40536, USA; menghanwang815@gmail.com (M.W.); astro11@email.uky.edu (A.S.); 2 Department of Molecular & Cellular Biochemistry, University of Kentucky, Lexington, KY 40508, USA; yanqi.xie@uky.edu (Y.X.); chunming.liu@uky.edu (C.L.); 3 Markey Cancer Center, University of Kentucky, Lexington, KY 40536, USA; jinpeng.liu@uky.edu (J.L.); li.chen@uky.edu (L.C.); smarno0@email.uky.edu (S.M.A.); 4 Division of Cancer Biostatistics, Department of Internal Medicine, University of Kentucky, Lexington, KY 40536, USA; 5 Department of Computer Science, Princeton University, Princeton, NJ 08540, USA; liaustintianli@gmail.com; 6 Division of Medical Oncology, Department of Internal Medicine, University of Kentucky, Lexington, KY 40536, USA

**Keywords:** cancer mutations, pathway analysis, intra-tumor heterogeneity

## Abstract

**Simple Summary:**

Cancer arises through the accumulation of somatic mutations in key biological pathways. This paper aims to develop a probabilistic approach to delineate the temporal order of mutations during cancer development based on mutation profile data from a cohort of patients. A unique feature of our method is that it incorporates intra-tumor heterogeneity (ITH) information, which refers to the heterogeneous cell populations within a tumor and characterizes the evolutionary history of the tumor. We showed that by integrating ITH, pathways, and functional annotation information, our method yielded high accuracy in inferring the temporal order of pathway mutations during carcinogenesis.

**Abstract:**

The development of cancer involves the accumulation of somatic mutations in several essential biological pathways. Delineating the temporal order of pathway mutations during tumorigenesis is crucial for comprehending the biological mechanisms underlying cancer development and identifying potential targets for therapeutic intervention. Several computational and statistical methods have been introduced for estimating the order of somatic mutations based on mutation profile data from a cohort of patients. However, one major issue of current methods is that they do not take into account intra-tumor heterogeneity (ITH), which limits their ability to accurately discern the order of pathway mutations. To address this problem, we propose PATOPAI, a probabilistic approach to estimate the temporal order of mutations at the pathway level by incorporating ITH information as well as pathway and functional annotation information of mutations. PATOPAI uses a maximum likelihood approach to estimate the probability of pathway mutational events occurring in a specific sequence, wherein it focuses on the orders that are consistent with the phylogenetic structure of the tumors. Applications to whole exome sequencing data from The Cancer Genome Atlas (TCGA) illustrate our method’s ability to recover the temporal order of pathway mutations in several cancer types.

## 1. Introduction

It is well understood that a human tumor is developed through the accumulation of somatic mutations in a number of essential biological pathways [1]. Studying the temporal order of somatic mutation occurrences is critically important to demystifying the biological mechanisms of tumor development, creating new therapeutic strategies, and predicting cancer prognosis. The most well-studied example of the order of mutations is from colorectal cancer, where the progression from normal epithelium to colorectal cancer often begins with mutations impacting the Wnt signaling pathway and subsequently progress with additional mutations in genes associated with MAPK, PI3K, TGF-beta, and p53 signaling pathways [1]. However, the temporal orders of mutations remain predominantly unknown in biological literature for many other types of cancer.

High-throughput DNA sequencing-based profiling of somatic mutations has presented an unparalleled opportunity to utilize statistical and computational methods in the study of cancer progression. Several methods have been devised to infer the order of somatic mutations based on mutation profile data from a cohort of patients. Fearon et al. modeled the relationship between mutations as a linear path [2]. Desper et al. [3] developed an oncogenetic tree model that illustrated the accumulation of mutations under ordering constraints by a tree. Szabo et al. [4] extended the pure oncogenetic tree model by introducing false positive and false negative observations, thereby increasing the model’s adaptability to accounting for potential sources of error. Beerenwinkel et al. [5] suggested using mixtures of oncogenetic trees, which offers a more adaptable option than fitting a single tree. Further, a more flexible conjunctive Bayesian network method was proposed [6,7,8], which did not presume a tree-like dependency structure and instead used a partially ordered set of mutations to describe the tumor’s evolutionary process. The aforementioned methods are topology-based, aiming to estimate the dependency structures among mutational events. Alternatively, Youn et al. [9] introduced a probabilistic approach to directly estimate the order of mutations without explicitly modeling the dependency structure. Instead, at each temporally ordered mutational step, they directly estimated the probability of each mutation occurring at that step. Wang et al. [10] extended this idea by incorporating functional annotation information of mutations and pathway annotations to improve the inference. These methods are free of complex modeling assumptions and, thus, are more robust and powerful in identifying early versus late mutations [11].

**Figure 1:**
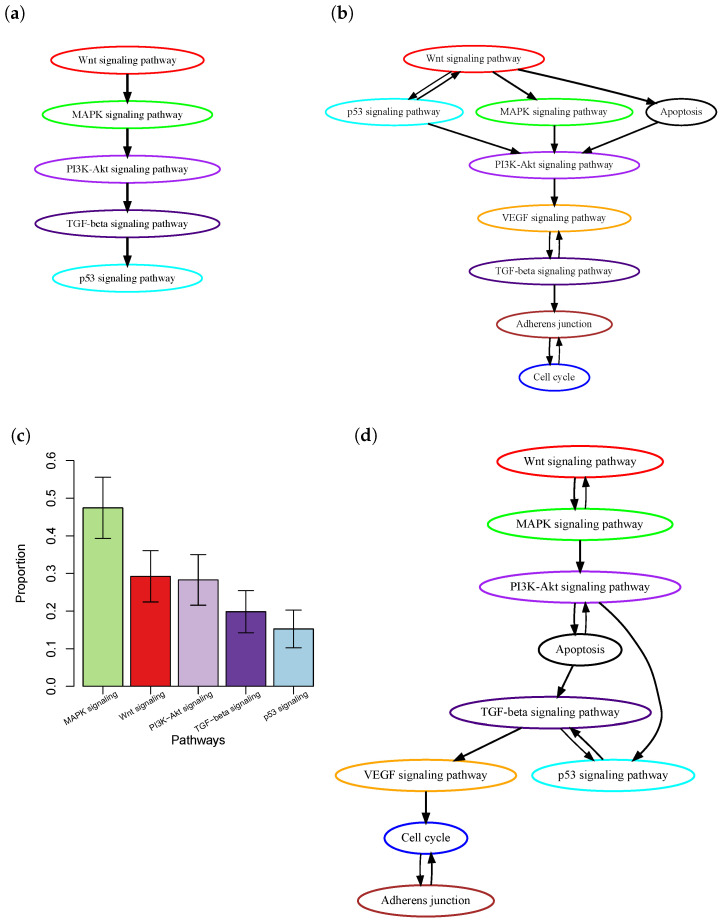
Order of pathway mutations in colon cancer. (**a**) The well-documented order of five key colon cancer pathways in biological literature [1]. (**b**) Order estimated based on the TCGA colon cancer dataset using the method by Wang et al. [10]. The figure was adapted from Figure 5 in [10] with modifications. (**c**) Clonality of mutations in those pathways. The y-axis is the proportion of at least one mutation of a pathway that is clonal, i.e., in the trunk of a phylogenetic tree. Results were obtained based on the TCGA colon cancer dataset using PhyloWGS [14]. Mean ± SEM are presented. (**d**) Order estimated based on the TCGA colon cancer dataset using PATOPAI.

One major limitation of current methods is that most of them only consider the presence or absence of mutations in a patient’s tumor, but do not take into account intra-tumor heterogeneity (ITH) [12,13]. ITH refers to the presence of a heterogeneous collection of cells, i.e., subclones, with distinct mutation profiles within a patient’s tumor. During cancer progression, new somatic mutations are continuously acquired by cancer cells. Some advantageous mutations, which provide cells with new functional characteristics, lead to the expansion of the cells to form intra-tumor subclones. The lack of accounting for ITH may lead to inaccurate inference of the order of pathway mutations. Consider the case of colon cancer, where the order of pathway mutations has been well-documented in the biological literature based on ample evidence from biological, pathological, and epidemiological studies [1] (Figure 1a). Using this known order of pathway mutations as a benchmark, we evaluated the order of pathway mutations inferred by Wang et al. [10] (Figure 1c) based on The Cancer Genome Atlas (TCGA) colon cancer whole-exome sequencing data. The p53 signaling pathway was reported to be mutated in a later stage of cancer development [1]. However, it was estimated to occur at an early stage; see the work by Wang et al. [10]. Further investigation into this discrepancy found that although many mutations occurred in the p53 signaling pathway, most of them were subclonal mutations (Figure 1b) that were expected to occur in the later stage of cancer development. Therefore, simple counting of the presence or absence of mutations will ignore such information and lead to inaccurate inference.

Several bioinformatic tools, such as PhyloWGS (v1.0) [14], Canopy (v1.3.0) [15], ClonEvol (v0.99.11) [16], SPRUCE (v0.99.0) [17] and PACTION (https://github.com/elkebir-group/paction) (accessed on 9 June 2024) [18], have been developed to infer the ITH information from either single-/multi-region bulk sequencing or single-cell sequencing. These tools usually characterize ITH by a phylogenetic tree or a set of trees with associated posterior probabilities, with nodes in the trees indicating different subclones and edges indicating the evolutionary relationships of subclones. As the phylogenetic trees describe the temporal order of mutations within an individual patient’s tumor, incorporating such in-depth intra-patient information into the tumor progression analysis across a cohort of patients is likely to substantially increase the power and accuracy of the analysis.

In this paper, we propose PATOPAI, a probabilistic model-based approach to characterize the temporal order of pathway mutations. PATOPAI incorporates the ITH information of each tumor sample by focusing the model on mutational sequences that are consistent with the phylogenetic structures of the sample. The pathway and functional annotation information of mutations is also integrated to enhance the inference. We applied PATOPAI to whole exome sequencing datasets of several cancer types from TCGA to demonstrate the performance of PATOPAI for delineating the order of pathway mutations.

## 2. Materials and Methods

### 2.1. Overview of the Method

**Figure 2:**
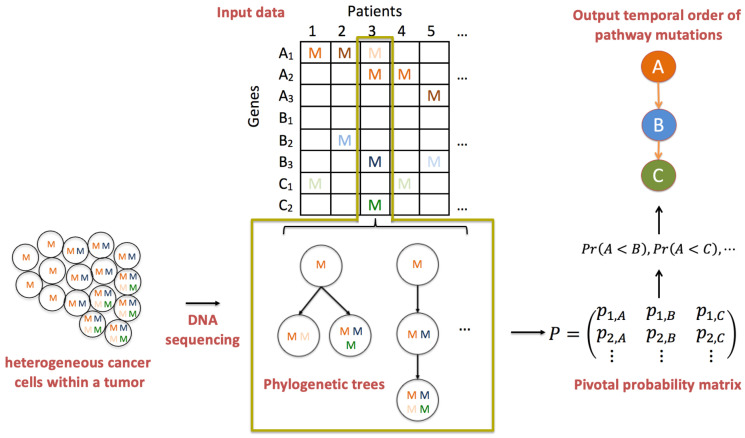
Overview of PATOPAI algorithm. Consider three pathways: *A* (having genes $A_{1}$, $A_{2}$, and $A_{3}$), *B* (having genes $B_{1}$, $B_{2}$, and $B_{3}$) and *C* (having genes $C_{1}$ and $C_{2}$). Letter “M” indicates observed mutation with a darker color indicating the mutation is more likely to be functional. For each patient’s tumor, which is a collection of heterogeneous cancer cells, the mutation profile is obtained based on DNA sequencing. Phylogenetic trees are then constructed to characterize the ITH of the tumor. Next, the ITH structure is integrated with pathway information and functional annotations to estimate a pivotal probability matrix *P*, where the (k,i) element of *P* is the probability that the *k*-th functional mutation occurs in the *i*-th pathway. Next, for each pair of pathways, we calculate the probability of one pathway being altered before the other, e.g., P(A<B) is the probability that *A* is mutated before *B*. Finally, a partial-order plot is generated to present the temporal order of pathway alterations.

An overview of PATOPAI is depicted in Figure 2. Our algorithm starts with three pieces of information from a cohort of patients at different stages of a specific cancer type. The first piece of information is a phylogenetic tree or a set of trees with associated posterior probabilities, which characterizes the ITH of each tumor sample. The second piece of information is the functional annotation information for each non-synonymous somatic mutation in a phylogenetic tree, which represents the likelihood of the mutation being functional. The third piece of information is the gene-to-pathway mapping for each mutation. We will take such data as the inputs for PATOPAI to delineate the common temporal order of pathway mutations across the cohort of patients based on a probabilistic method. The primary part of PATOPAI is to estimate a pivotal probability matrix *P*, where the (k,i) element of *P* indicates the probability of the *k*-th functional mutation occurring in the *i*-th pathway. Utilizing *P*, we calculate the probability of one pathway acquiring alterations before or after another for each pair of pathways. Lastly, a simplified partial-order graph is employed to summarize the results and visualize the temporal order of all pathways.

The fundamental concept of PATOPAI can be elucidated through the example of delineating the temporal order of pathways A, B, and C as depicted in Figure 2. First, observe that every patient with mutations in pathway B also exhibits mutations in pathway A. Conversely, some patients (patients 2 and 5) only have mutations in pathway A without mutations in pathway B. This implies that pathway A is likely to acquire mutations before pathway B in the process of tumorigenesis. This concept originated from the work of Youn and Simon [9], and was extended by Wang et al. [10] to incorporate functional annotation information and perform analysis at the pathway level instead of individual genes to improve the inference. Figure 2 highlights the importance of further incorporating ITH information in such analysis. For example, patient 3 has mutations in all three pathways. Without studying ITH, data from this patient lacks the informativeness required to determine the order of the three pathways. However, phylogenetic trees of patient 3 suggest that the mutation in pathway A occurs first in the patient, followed by mutations in pathways B and C. Including this additional ITH information renders the data from this patient useful in substantiating the idea that pathway A is altered before pathways B and C. Figure 2 also illustrates the use of pathway analysis and functional annotation information. For example, it is difficult to infer the order of $A_{3}$ and $B_{2}$ since each gene only has one mutation that resides in different patients. In contrast, the mutation frequency at the pathway level is much higher, so estimating the temporal order between pathways A and B is more feasible. As another example, mutations are present in both pathways A and C in patient 5. Using functional annotation information, the mutation in pathway A is more likely to be functional, while the one in pathway C is more likely to be non-functional, which provides useful information supporting that A is altered before C. Thus, the integration of ITH, pathway, and functional annotation information will substantially increase the accuracy of cancer progression inference.

### 2.2. Probability Model

Let $Y_{i}^{j}$ represent the count of non-synonymous mutations in pathway *i*(i=1,…,N), and $c_{j}$ represent the total count of non-synonymous mutations in patient *j*(j=1,…,M). Define $S_{k}^{j}$ to indicate whether the *k*-th mutation is functional ($S_{k}^{j}$ = 1) or not ($S_{k}^{j}$ = 0) for $k=1,\ldots ,c_{j}$, and let $n_{j}=\sum_{k=1}^{c_{j}}S_{k}^{j}$ be the total count of functional mutations in patient *j*. To consider ITH, let $T_{j}$ represent the phylogenetic tree of patient *j*, which takes a number of candidate tree structures with their associated posterior probabilities obtained from software such as PhyloWGS [14]. According to the law of total probability, the likelihood of observing $\left(Y_{1}^{j},\ldots ,Y_{N}^{j}\right)$ can be formulated as follows:

(1) $$\begin{array}{cc} \; & P(Y_{1}^{j},\ldots ,Y_{N}^{j})\\ = & \sum_{t}\sum_{s_{1},\ldots s_{c_{j}}}P\left(Y_{1}^{j},\ldots ,Y_{N}^{j}|T_{j}=t,S_{1}^{j}=s_{1},\ldots ,S_{c_{j}}^{j}=s_{c_{j}},c_{j}\right)\\ & \;\times P\left(S_{1}^{j}=s_{1},\ldots ,S_{c_{j}}^{j}=s_{c_{j}}|T_{j}=t,c_{j}\right)P\left(T_{j}=t|c_{j}\right)P\left(c_{j}\right), \end{array}$$

where the first summation is over all phylogenetic trees for patient *j* and the second summation encompasses all possible sequences of $S_{1}^{j},\ldots ,S_{c_{j}}^{j}$. Next, we explain the computation of each of the four terms in the summation. Starting with the first term, let $D_{k}^{j}$ represent the (unknown) identity of the pathway that was mutated as the *k*-th functional event, and $N_{l}^{j}$ represent the (unknown) identity of the pathway that was mutated as the *l*-th non-functional event in patient *j*. For any specific phylogenetic tree $T_{j}=t$, there may be multiple orders of occurrence of $D_{k}^{j}$ and $N_{l}^{j}$ that are consistent with tree *t*. An order of pathway identities of mutations, $\left(i_{1},\ldots ,i_{K}\right)$, is considered consistent with *t* if there exists a sequence of mutations, $\left(m_{1},\ldots ,m_{K}\right)$, from *t*, such that $m_{k}$ belongs to pathway $i_{k}$ for k=1,…,K, and $m_{u}$ is not downstream of $m_{v}$ in tree *t* for any u<v. Let $D_{t}=\left\{\left(i_{1},\ldots ,i_{n_{j}}\right)|\left(i_{1},\ldots ,i_{n_{j}}\right)\mathrm{is}\mathrm{consistent}\mathrm{with}t\right\}$ and $N_{t}=\left\{\left(i_{n_{j}+1},\ldots ,i_{c_{j}}\right)|\left(i_{n_{j}+1},\ldots ,i_{c_{j}}\right)\mathrm{is}\mathrm{consistent}\mathrm{with}t\right\}$ be the sets of all potential sequences of pathway identities for functional and non-functional mutations, respectively. We have the following:

(2) $$\begin{array}{cc} \; & \;P(Y_{1}^{j},\ldots ,Y_{N}^{j}|T_{j}=t,S_{1}^{j},\ldots S_{c_{j}}^{j},c_{j})\\ \; & \;=\sum_{\begin{array}{c} \left(i_{1},\ldots ,i_{n_{j}}\right)\in D_{t}\\ \left(i_{n_{j}+1},\ldots ,i_{c_{j}}\right)\in N_{t} \end{array}}P\left(D_{1}^{j}=i_{1},\ldots ,D_{n_{j}}^{j}=i_{n_{j}},N_{1}^{j}=i_{n_{j}+1},\ldots ,N_{c_{j}-n_{j}}^{j}=i_{c_{j}}|T_{j}=t,S_{1}^{j},\ldots ,S_{c_{j}}^{j},c_{j}\right)\\ \; & \;=\sum_{\begin{array}{c} \left(i_{1},i_{2},\ldots ,i_{n_{j}}\right)\in D_{t}\\ \left(i_{n_{j}+1},\ldots ,i_{c_{j}}\right)\in N_{t} \end{array}}P\left(D_{1}^{j}=i_{1},\ldots ,D_{n_{j}}^{j}=i_{n_{j}}|T_{j}=t,S_{1}^{j},\ldots ,S_{c_{j}}^{j},c_{j}\right)\\ \; & \;\times P(N_{1}^{j}=i_{n_{j}+1},\ldots ,N_{c_{j}-n_{j}}^{j}=i_{c_{j}}|T_{j}=t,S_{1}^{j},\ldots ,S_{c_{j}}^{j},c_{j}), \end{array}$$

where the last equation is derived under the assumption of independence of occurrences between functional mutations and non-functional mutations. By further assuming that the occurrences of mutations are independent and they are independent of $c_{j}$, the probability for each possible sequence can be expressed as the product of the probabilities that $D_{k}^{j}=i_{k}$ and $N_{l}^{j}=i_{l}$. Denote $p_{k,i_{k}}$ as the probability that $D_{k}^{j}=i_{k}$. These $p_{k,i_{k}}$ values are parameters of interest, where we define the pivotal probability matrix as $P={\left(p_{k,i_{k}}\right)}_{K\times N}$. For non-functional mutations, we assume that all orders of non-functional mutations have the same probability as their occurrences are most likely random. Define $q_{i}$ as the probability that a non-functional mutation occurs in pathway *i*, we estimate it by calculating the expected fractions of non-functional mutations that are from pathway *i* across patients. Specifically, following Wang et al. [10], let $r_{k}=P\left(S_{k}^{j}=1\right)$ be the functional impact score of the *k*th mutation. The expected number of non-functional mutations in pathway *l* for patient *j* is $E_{l}^{j}=\sum_{k:\mathrm{the}k\mathrm{th}\mathrm{mutation}\mathrm{is}\mathrm{from}\mathrm{pathway}l}\left(1-r_{k}\right)$. We can then estimate $q_{i}$ by $\left(\sum_{j}E_{i}^{j}\right)/\left(\sum_{j}\sum_{l}E_{l}^{j}\right)$, an average of $E_{l}^{j}$ across patients. Assuming that all mutations are independent, the above probability can be calculated as follows:

(3) $$\sum_{\begin{array}{c} \left(i_{1},i_{2},\ldots ,i_{n_{j}}\right)\in D_{t}\\ \left(i_{n_{j}+1},\ldots ,i_{c_{j}}\right)\in N_{t} \end{array}}\left(\prod_{k=1}^{n_{j}}p_{k,i_{k}}\right)\left(\prod_{l=n_{j}+1}^{c_{j}}q_{i_{l}}\right).$$

For the second and fourth terms in Equation (1), the assumptions and calculations are identical to what was performed in our prior paper [10]. For the third term, $P(T_{j}|c_{j})$ is obtained from software such as PhyloWGS [14] or Canopy [15]. These software are designed to reconstruct the evolutionary history of a tumor based on bulk DNA sequencing data. They characterize subclones and delineate the phylogeny within a tumor using phylogenetic trees. The outputs are possible phylogenetic tree structures of a tumor and their associated posterior probabilities, which are used to specify $P(T_{j}|c_{j})$ in our method. Therefore, the likelihood function can be written as follows:

(4) $$\begin{array}{c} \begin{array}{c} \;\prod_{j}\sum_{T_{j}}P\left(T_{j}|c_{j}\right)\left\{\sum_{S_{1}^{j},\ldots ,S_{c_{j}}^{j}}\left(\sum_{\begin{array}{c} \left(i_{1},i_{2},\ldots ,i_{n_{j}}\right)\in D_{t}\\ \left(i_{n_{j}+1},\ldots ,i_{c_{j}}\right)\in N_{t} \end{array}}\left(\prod_{k=1}^{n_{j}}p_{k,i_{k}}\right)\left(\prod_{l=n_{j}+1}^{c_{j}}q_{i_{l}}\right)\right)\right\}\left\{\prod_{k=1}^{c_{j}}r_{k}^{S_{k}^{j}}{\left(1-r_{k}\right)}^{1-S_{k}^{j}}\right\}. \end{array} \end{array}$$

Finally, in accordance with Wang et al. [10], we calculate P(A<B), the probability that pathway *A* is altered before pathway *B* is altered, as

(5) $$P\left(A<B\right)=\Sigma_{\left\{\left(i_{1},\ldots ,i_{n_{j}}\right)\in G_{A<B}\right\}}\left(\prod_{k=1}^{c}p_{k,i_{k}}\right),$$

where $c=\max_{j\in \{1,\ldots ,M\}}\left\{c_{j}\right\}$ and $G_{A<B}$ is the collection of pathway mutation sequences satisfying the first functional mutation in pathway *A* precedes the first functional mutation in *B*.

It is important to note that the method assumes non-overlapping pathways. In cases where pathways have overlapping genes, we re-organize the genes into sets that are mutually exclusive to perform the analysis and compute three probabilities, P(A<B), P(B<A), and P(A=B), as described in [10]. The results obtained were visualized with a simplified version of a partial-order plot [19]. Specifically, each node in the plot represents a pathway. The thickness of a directed edge from one pathway to another indicates the estimated temporal-order probability between the two pathways. The nodes are arranged based on the layered graph drawing method in Graphviz [20] according to the estimated temporal order of pathways. For clarity, an edge with probability below a certain threshold is eliminated from the plot. An edge between two pathways that are indirectly connected by other edges is also removed, e.g., the edge from *A* to *C* is removed if there is an edge from *A* to *B* and an edge from *B* to *C*.

### 2.3. Entropy Constraint

A key step in our method is to estimate the pivotal probability matrix *P*. Due to its high dimensionality, the maximum likelihood estimate can be unstable. We address this issue by introducing the following entropy constraint to regularize the estimation. It is natural to expect that the certainty for delineating the mutational order of pathways is the highest at the first functional mutational event, and decreases as more mutational events occur. Based on information theory, the level of uncertainty for the *k*-th row of *P* matrix is quantified by entropy [21], $-\sum_{v}p_{k,v}\log\left(p_{k,v}\right)$. Therefore, we consider the following entropy constraint:

(6) $$\begin{array}{c} -\sum_{i_{k_{1}}}p_{k_{1},i_{k_{1}}}\log\left(p_{k_{1},i_{k_{1}}}\right)<-\sum_{i_{k_{2}}}p_{k_{2},i_{k_{2}}}\log\left(p_{k_{2},i_{k_{2}}}\right),\mathrm{for}\forall \;k_{1}<k_{2}. \end{array}$$

An estimate of $p_{k,i_{k}}$ is obtained by maximizing the likelihood under the entropy constraint. In addition to the entropy constraint, we also implement several approaches to improve the computational efficiency of the estimation, which are described in the following subsection.

### 2.4. Computational Efficiency Optimization

The estimation of the pivotal probability matrix *P* is computationally intensive. The number of parameters in *P* increases with both the number of pathways and the number of mutations. In addition, recent phylogenetic analysis tools like PhyloWGS [14] output a large number (several thousand) of candidate trees from the posterior distribution over phylogenies for each patient. The large number of trees causes a heavy computational burden. Further, the computational complexity for finding all the possible orders that are consistent with each phylogenetic tree increases sharply as the number of mutations used to construct the tree increases.

To improve the computational efficiency of our method, we consider the following three approaches. First, we conduct our analysis independently for each pair of pathways rather than analyzing all pathways together. This approach decreases the number of parameters we need to estimate at one time. It also reduces the complexity of finding the orders that are consistent with a phylogenetic tree since we only need to focus on the limited number of mutations belonging to the two pathways under consideration. This pairwise approach has been shown to yield similar results as the approach of analyzing all pathways together [10]. Second, as another advantage of the pairwise approach, we find that many candidate trees of a patient reduce to be the same when we confine the trees to only include mutations in a pair of pathways. Thus, for each pair of pathways, we combine trees with the same structure for mutations in the two pathways into one reduced tree with a posterior probability equal to the summation of posterior probabilities of the original trees. We then sort the reduced trees in descending order of their posterior probabilities, only considering the top ones whose cumulative probability reaches 95%. Based on this approach, the number of trees to be computed for each patient is largely reduced. Third, we reduce the number of parameters by setting $P_{k,i}=P_{k_{0},i}$ for $k>k_{0}$, which represents an averaged distribution for mutations occurring after the $k_{0}$-th step. Wang et al. [10] showed that the estimated probabilities stabilize for $k_{0}\geq 4$. Thus, we set $k_{0}=4$ in our analyses.

## 3. Results

To assess the performance of PATOPAI, we utilized it to analyze whole exome sequencing data from TCGA for colon cancer (*n* = 461), hepatocellular carcinoma (*n* = 377), glioblastoma multiforme (*n* = 617), and pancreatic adenocarcinoma (*n* = 185). For each cancer type, we selected the key cancer pathways according to the KEGG database [22]. The genes within each pathway, as listed in Supplementary Table S1, were identified using the KEGG database with manual curation. In our analyses, phylogenetic trees of each tumor sample were obtained using PhyloWGS [14]. Data analysis was performed on the Morgan Compute Cluster of the University of Kentucky. Each compute node consists of an AMD 7702P CPU, with cores and 512 GB RAM. For each pair of pathways, the analysis was performed with 10 CPU cores and 64 GB of RAM. In this setup, the running time for these tasks varies between 220 s and 506 min, depending on the number of mutations and complexities of tree structures.

### 3.1. Analysis of TCGA Colon Adenocarcinoma Data

The TCGA-COAD project dataset contains whole exome sequencing data of tumor samples from 461 colon cancer patients. We excluded the top 16% of hyper-mutated samples, as the process of tumorigenesis in these samples involves specific sequences of genetic events [23], and the remaining 391 samples were used in our analysis. The temporal-order probabilities for each pair of pathways were estimated by PATOPAI, and the results were summarized in a simplified partial-order plot. (Figure 1d). Without considering the ITH information, Wang et al. [10] previously found that p53 pathway mutation occurred earlier than that of PI3K signaling mutations in colon cancer (Figure 1c). After incorporating the ITH information into the analysis, PATOPAI now shows that the pathway mutations in colon cancer follow a temporal order of WNT–MAPK–apoptosis–PI3K–p53–TGF-beta signaling pathways (Figure 1d), which agrees better with the ‘classic’ colorectal cancer formation model [1] since it puts p53 after the PI3K signaling pathway. The p53 pathway functions as a critical tumor suppressor mechanism and induces cell cycle arrest and apoptosis. Our analysis reveals that p53 pathway mutations occur after cancer cells accumulate pro-growth mutations in the WNT–MAPK–apoptosis–PI3K pathways. In late tumorigenesis processes, tumor cells initiate EMT and metastasize into other tissues, where TGF-beta–VEGF–adherens junction pathways play important roles [24]. Our analysis shows these pathways mutate late in the tumor formation process, which demonstrates improved predicting performance of our method.

### 3.2. Analysis of TCGA Hepatocellular Carcinoma Data

**Figure 3:**
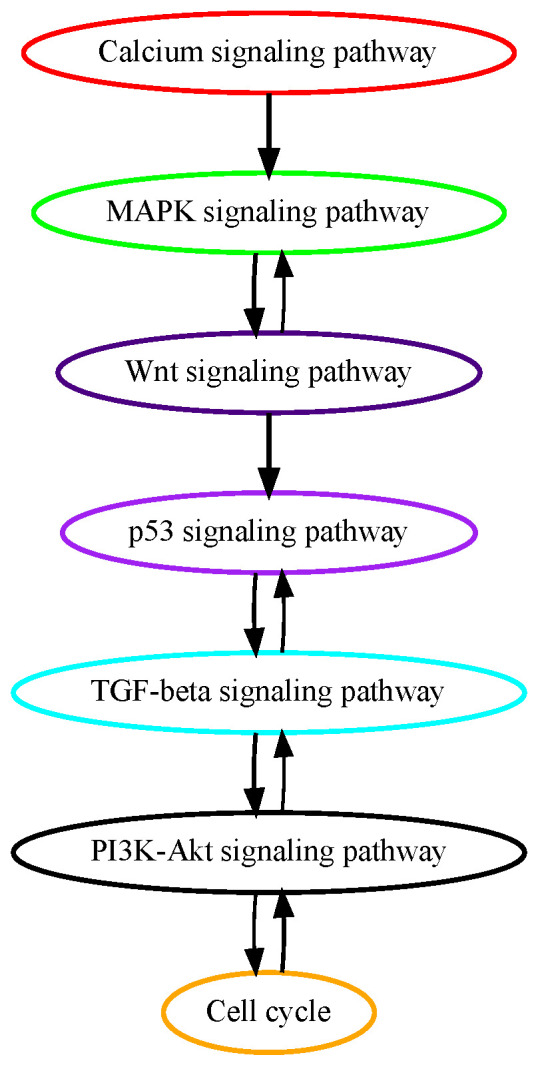
Estimated order of pathway mutations based on TCGA HCC dataset. Each node represents a pathway, with nodes/pathways arranged according to estimated temporal-order probabilities. The width of a directed edge is proportional to the probability of the head node pathway being mutated before the tail node pathway. To enhance visualization, edges with probabilities < 0.4 have been excluded. In addition, the direct edge between two pathways that are indirectly connected by other edges is also removed for clarity, e.g., for three pathways with the order of A→B→C, the edge A→C will be removed from this figure.

The pathogenesis of hepatocellular carcinoma (HCC) is primarily driven by genetic accumulation of molecular mutations governing oncogenic signaling pathways. Numerous alterations in genes in calcium signaling pathways have been identified in the early stages of HCC tumorigenesis [25]. Mutations in genes encoding proteins controlling calcium signaling processes including Ca^2+^ entry, release, and binding have been found and have profound implications in promoting the liver cancer cell cycle, metabolism, proliferation, and tumorigenesis in tumor initiations [25]. These previous findings confirm the results of our pathway mutation analysis that calcium signaling pathway mutations are on an earlier temporal order in HCC development (Figure 3). Around 50% of HCC cases harbor activating mutations in the MAPK signaling pathway, which, among other mutated signaling pathways in HCC, is deemed to be the most important in the HCC tumorigenesis process [26]. Aberrant activation of beta-catenin, which activates the WNT signaling pathway is observed in about 30% of HCC patients. The second most mutated gene in the WNT signaling pathway is AXIN1 which occurs in about 10% of HCC cases [27]. TP53 pathway mutations happen in around 30% of HCC patients and regulate gene expression in cancer cell apoptosis and senescence. Our results in pathway mutation analysis showed that these growth, proliferation, and apoptosis-related signaling pathways mutated in early to midterm stages in HCC.

### 3.3. Analysis of TCGA Glioblastoma Multiforme Data

**Figure 4:**
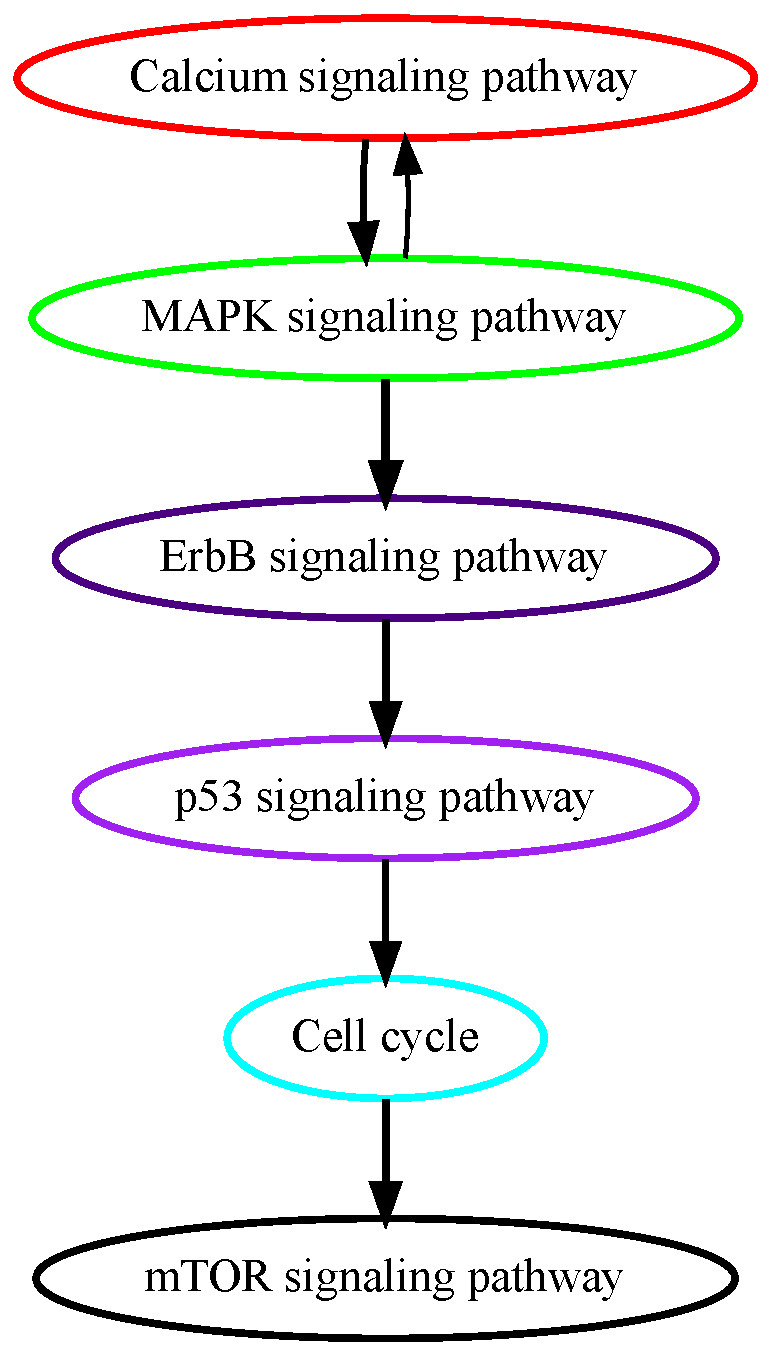
Estimated order of pathway mutations based on TCGA GBM dataset. Each node represents a pathway, with nodes/pathways arranged according to estimated temporal-order probabilities. The width of a directed edge is proportional to the probability of the head node pathway being mutated before the tail node pathway. To enhance visualization, edges with probabilities < 0.4 are excluded, and edges between pathways that are indirectly connected by other edges are also removed from this figure.

Glioblastoma multiforme (GBM) is grade IV glioma, the most aggressive malignant tumor in the central nervous system with patients’ mean survival time of less than 16 months [28]. The low-grade benign gliomas (Grade I) and lower-grade astrocytomas (Grade II/III) eventually develop into GBM through complicated processes in which a number of genetic alterations have been reported. While the mechanisms of how normal glial progenitor cells transform into low-grade gliomas are still under investigation, genetic alterations in calcium transportation and binding proteins in the calcium signaling pathway were found both in normal glial development and low-grade gliomas [29]. Ca^2+^, a second messenger that is involved in neuronal signaling, and other cellular processes, such as cell proliferation, apoptosis, and autophagy, may contribute to cancer development when dysregulated. TRPM7 (transient receptor potential cation channel subfamily M member 7) facilitates the transport of calcium ions, and its pharmacological inhibition decreases the proliferation, migration, and invasion of glioma cells [30]. These data suggest that alterations in calcium signaling occur at higher temporal orders in GBM progression, and our pathway analysis successfully captured calcium signaling as an early event (Figure 4). ErbB signaling pathway and its downstream mitogen-activated protein kinase (Ras/MAPK) signaling pathway regulate cellular processes such as growth, survival, differentiation, and migration and are frequently mutated in GBM [31]. A recent study analyzed genomic sequencing data from the temporally separated tumor pairs of 304 adult patients and found acquired CDKN2A deletions were associated with increased cell proliferation at tumor recurrence, suggesting mutations in cell cycle signaling might be late in temporal order in GBM development [32].

### 3.4. Analysis of TCGA Pancreatic Adenocarcinoma Data

**Figure 5:**
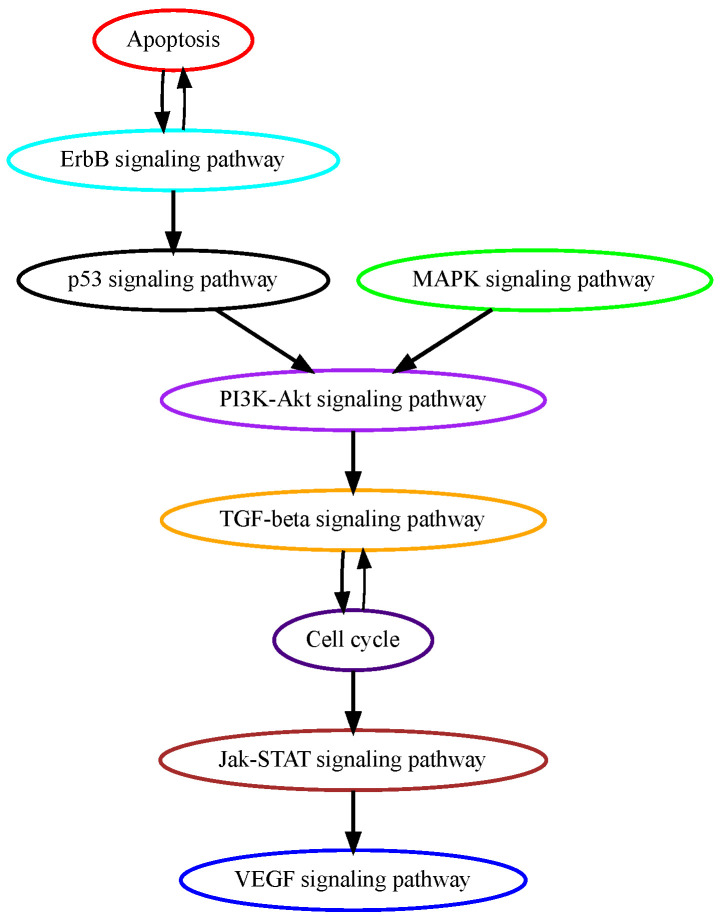
Estimated order of pathway mutations based on TCGA PAAD dataset. Each node represents a pathway, with nodes/pathways arranged according to estimated temporal-order probabilities. The width of a directed edge is proportional to the probability of the head node pathway being mutated before the tail node pathway. To enhance visualization, edges with probabilities < 0.4 are excluded, and edges between pathways that are indirectly connected by other edges are also removed from this figure.

Pancreatic adenocarcinoma (PAAD) remains one of the most fatal cancers with a 5-year survival rate of around 8% [33]. The genetic progression model of pancreatic carcinogenesis has largely been established. Generally, pancreatic duct epithelium transforms into three progressing stages of pancreatic intraepithelial neoplasia (PanIN): PanIN-1, PanIN-2, and PanIN-3, eventually developing into pancreatic cancer [34]. More than 90% of KRAS mutations are found in PanIN-1 lesions, suggesting that MAPK signaling mutations are on an early temporal order in the PAAD tumorigenesis process [35]. The tumor suppressor gene CDKN2A is commonly inactivated in pancreatic cancer and can be detected as early as PanIN-2 lesions [36]. In PanIN-3 lesions, inactivating mutations of TP53 and SMAD4 in TGF-beta signaling were found [37]. While our model correctly recognizes MAPK signaling as an early mutational event and TGF-beta signaling as a late event, it contradicts the ligature by placing cell cycle signaling at a lower temporal position (Figure 5). Other mutations in the cycle cell signaling pathway may be late events but CDKN2A mutations happen as early as in PanIN-2 lesions.

### 3.5. Comparison to Other Methods

We compared PATOPAI with two existing methods, OncoTree (2002) [4] and the method of Youn and Simon (2012) [9]. We focused our comparison on colon cancer, as for this cancer type, the temporal order of pathway alterations is well-documented in biological literature [1]; see Figure 1a. The estimated oncogenetic tree obtained from the R package OncoTree is provided in Figure S1. OncoTree was able to infer that the MAPK signaling pathway was altered before PI3K, p53, and TGF-beta signaling pathways, but failed to determine the Wnt signaling pathway as an upstream altered event. Also, it was unable to determine the order between the p53 and PI3K/TGF-beta signaling pathways. The result obtained from the method of Youn and Simon is provided in Figure S2. This method estimated that the first event most likely occurred in the MAPK signaling pathway and the fourth event occurred in the Wnt signaling pathway, which was inconsistent with the biological literature that the Wnt signaling pathway was altered before the MAPK signaling pathway. Likewise, it was also unable to determine the correct order between the PI3K and TGF-beta signaling pathways, as it inferred that the second event most likely occurred in the TGF-beta signaling pathway and the fourth event had a high probability of occurring in the PI3K signaling pathway. Therefore, the results from both methods did not align as closely with previous biological evidence compared to our method.

#### Comparison of Computational Efficiency

We evaluated the performance of our proposed computational efficiency optimization approaches. Our evaluation was based on estimating the temporal order of mutations in the MAPK and TGF-beta signaling pathways, using the TCGA pancreatic adenocarcinoma data. The running time with our computational efficiency optimization was 371 s. Without setting $P_{k,i}=P_{k_{0},i}$ for $k>k_{0}$, the running time of the same task increased to 430 s. By further excluding the approach of combining trees with the same structures in our method, the running time increased to 732 s. Therefore, our computational efficiency optimization shortened the computational time by half in this case.

## 4. Discussion

Our analysis focuses on delineating the common temporal order of pathway mutations across a cohort of patients by leveraging ITH, which provides the cancer progression information within each individual patient’s tumor, along with pathway and mutational functional annotation information. In our application studies, we used phylogenetic trees obtained from PhyloWGS [14] to characterize the ITH information. Other methods, such as Canopy [15], ClonEvol [16], and SPRUCE [17], can also be used as alternatives. Note that in our analysis, the ITH information is obtained from bulk sequencing as such data are widely available. But our method can also take ITH information from single-cell DNA sequencing, which quantifies ITH at a much higher resolution. One may replace PhyloWGS with phylogenetic analysis methods for single-cell data such as SCITE [38], SciFit [39], and SiCloneFit [40] to characterize the ITH of individual tumors, and then use the ITH for each individual patient’s tumor as an input for our method to infer the common temporal order of mutations across a cohort of patients. Note that for a phylogenetic tree inferred by SciFit or SiCloneFit, the temporal order of mutations can be obtained by inferring the mutation status of internal nodes based on a maximum likelihood approach as described in SciFit and SiCloneFit papers [39,40]. Ultimately, we anticipate our method could also be applied in the future using single-cell DNA sequencing data of a cohort of patients once such data becomes more accessible.

One limitation of our analysis is that we did not account for cancer subtypes [41,42]. As cancer is a complex disease, tumors of similar morphology often have dramatically different outcomes and responses to treatment [43,44]. Cancer sub-typing plays an important role in cancer prognosis and more personalized treatment planning. Analyzing each cancer subtype and comparing the results across subtypes would be intriguing. Such an approach may facilitate the identification of both common and subtype-specific mutational orders and provide new insights into the biological mechanisms of cancer development and drug resistance. This will be one of the crucial focuses in our future work.

## 5. Conclusions

In this paper, we introduced a novel model to estimate the temporal order of pathway mutations by integrating ITH, pathways, and mutational functional annotation information. We applied our method to studying the progression of colon, liver, glioblastoma, and pancreatic cancers at the level of biological pathways. The results show that our method yielded high accuracy for the cancer progression inference. Our analysis demonstrates that the ITH information is critical to recovering the temporal order of pathway mutations from cross-sectional datasets.

## Data Availability

Our method is freely available for academic use at https://github.com/MarkeyBBSRF/PATOPAI, accessed on 9 June 2024.

## Supplementary Materials

The following supporting information can be downloaded at https://www.mdpi.com/article/10.3390/cancers16132488/s1. Table S1: “Core” Pathway genes; Figures S1 and S2: Estimated orders of pathway mutations based on the TCGA colon cancer dataset using OncoTree and Youn’s method, respectively.

## Abbreviations

The following abbreviations are used in this manuscript:

ITH	intra-tumor heterogeneity
PATOPAI	a probabilistic approach for estimating the temporal order of pathway mutations by incorporating ITH information
TCGA	The Cancer Genome Atlas
HCC	hepatocellular carcinoma
GBM	glioblastoma multiforme
PAAD	pancreatic adenocarcinoma
