# Supplementary material for: A Probabilistic Approach to Estimate the Temporal Order of Pathway Mutations Accounting for Intra-Tumor Heterogeneity
Source: Cancers (Basel). 2024 Jul 8;16(13):2488. doi: 10.3390/cancers16132488 (PMC11240401; doi:10.3390/cancers16132488)
Supplement: Supplementary file 1 [file cancers-16-02488-s001.zip › supplementary_figures.pdf]

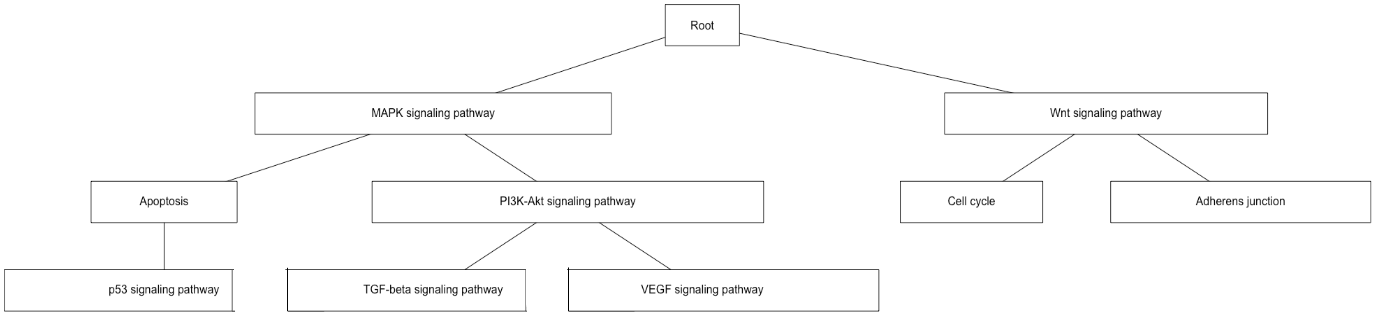

Figure S1: **Temporal orders of colon cancer inferred by R package Oncotree.** For methods comparison, we applied R package Oncotree (version 0.3.5)[1] to TCGA colon cancer data. We used the 'oncotree.fit' function with default parameter settings to obtain the estimated oncogenetic tree, which indicates the dependency orders of pathway mutations. The observed mutation profiles at the gene level are converted into a list of altered core pathways, based on the assumption that a pathway is considered altered if at least one of its genes is mutated.

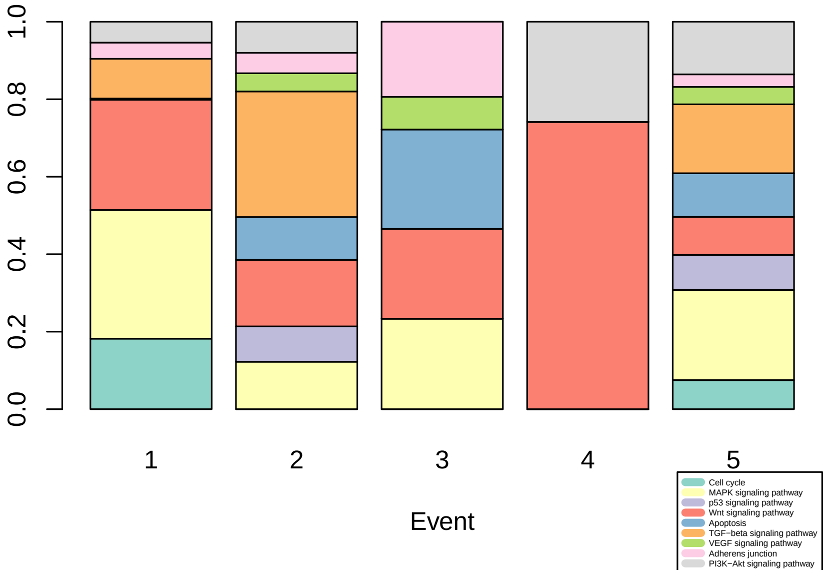

Figure S2: **Temporal orders of colon cancer inferred by Youn's method.** For methods comparison, we applied Youn's method[2] to TCGA colon cancer data. We set K=4 and N=10 to obtain the estimated probability matrix, and used a bar plot to display the results, as Youn did in the original paper. The observed mutation profiles at the gene level were converted into pathway-level mutation profiles by summing the number of mutations of all mutated genes within each pathway.

[1] Aniko Szabo and Kenneth Boucher. Estimating an oncogenetic tree when false negatives and positives are present. *Mathematical biosciences*, 176(2):219–236, 2002.
[2] Ahrim Youn and Richard Simon. Estimating the order of mutations during tumorigenesis from tumor genome sequencing data. *Bioinformatics*, 28(12):1555–1561, 2012.
